# Deep Intronic LINE-1 Insertions in *NF1*: Expanding the Spectrum of Neurofibromatosis Type 1-Associated Rearrangements

**DOI:** 10.3390/biom13050725

**Published:** 2023-04-23

**Authors:** Viola Alesi, Silvia Genovese, Francesca Romana Lepri, Giorgia Catino, Sara Loddo, Valeria Orlando, Silvia Di Tommaso, Alessandra Morgia, Licia Martucci, Maddalena Di Donato, Maria Cristina Digilio, Bruno Dallapiccola, Antonio Novelli, Rossella Capolino

**Affiliations:** 1 Laboratory of Medical Genetics, Translational Cytogenomics Research Unit, Bambino Gesù Children Hospital, IRCCS, 00146 Rome, Italy; 2 Medical Genetics Unit, Bambino Gesù Children Hospital, IRCCS, 00146 Rome, Italy; 3 Genetics and Rare Disease Research Division, Bambino Gesù Children Hospital, IRCCS, 00146 Rome, Italy

**Keywords:** neurofibromatosis type 1, *NF1*, optical genome mapping, whole genome sequencing, LINE-1 element, transposable elements

## Abstract

Neurofibromatosis type 1 is an autosomal-dominant condition caused by *NF1* gene inactivation. Clinical diagnosis is corroborated by genetic tests on gDNA and cDNA, which are inconclusive in approximately 3–5% of cases. Genomic DNA approaches may overlook splicing-affecting intronic variants and structural rearrangements, especially in regions enriched in repetitive sequences. On the other hand, while cDNA-based methods provide direct information about the effect of a variant on gene transcription, they are hampered by non-sense-mediated mRNA decay and skewed or monoallelic expression. Moreover, analyses on gene transcripts in some patients do not allow tracing back to the causative event, which is crucial for addressing genetic counselling, prenatal monitoring, and developing targeted therapies. We report on a familial NF1, caused by an insertion of a partial LINE-1 element inside intron 15, leading to exon 15 skipping. Only a few cases of LINE-1 insertion have been reported so far, hampering gDNA studies because of their size. Often, they result in exon skipping, and their recognition of cDNA may be difficult. A combined approach, based on Optical Genome Mapping, WGS, and cDNA studies, enabled us to detect the LINE-1 insertion and test its effects. Our results improve knowledge of the NF1 mutational spectrum and highlight the importance of custom-built approaches in undiagnosed patients.

## 1. Introduction

Neurofibromatosis type 1 (NF1) is an autosomal dominant condition caused by inactivating variants in the *NF1* gene. The associated phenotype is gradually progressive, presenting with full penetrant and highly variable intrafamilial expression. Clinical manifestation is characterized by skin pigmentary lesions, multiple cutaneous neurofibromas, and, less frequently, brain and peripheral nerve tumors, skeletal abnormalities, learning disabilities, and behavioral problems. Clinical diagnosis is challenging due to the heterogeneity of distinguishing features and their age-related occurrence, often hampering a timely diagnosis. Molecular confirmation is crucial for addressing appropriate management, assessing the reproductive risk, prenatal monitoring, and developing targeted therapies [1].

Currently, the recommended molecular approach includes a multistep protocol on gDNA and cDNA, comprising NGS sequencing and MLPA (Multiplex Ligation-dependent Probe Amplification) assays, which are diagnostic in 95–97% of cases [2,3]. A small percentage of patients fulfilling the NF1 diagnostic criteria [4] remains molecularly undiagnosed due to mosaicisms or variants overlooked by standard techniques [5,6,7,8,9]. In fact, gDNA analysis may overlook deep intronic variants which, even when detected, often present with unpredictable effects on the gene function. Moreover, due to the technical limitation, gDNA techniques present a low resolution for structural rearrangements and large insertions, especially when involving highly repetitive regions.

On the other hand, providing direct information about gene transcription, cDNA analysis is often decisive in resolving interpretative doubts, but it may result in being inconclusive in the case of non-sense-mediated mRNA decay and skewed or mono-allelic expression of the *NF1* wild-type allele [6].

Transposable elements (TEs) integration (mostly Alu and LINE-1) has been reported in association with Neurofibromatosis type 1, accounting for approximately 0.4% of mutations in the disease gene [6,7] due to its AT-reach sequence, favoring retrotransposons integration. The frequency of TE as etiological factors in Neurofibromatosis 1 may be underestimated due to the detection methods used, which are usually based on the amplification of small amplicons. LINE-1 elements play an important role in evolution and are traceable in the human phylogenetic tree since eutherian mammalian ancestors [10]. Although they account for more than 17% of human DNA, the vast majority is currently unable to move along the genome, due to the accumulation of rearrangements, deletions, 5′ truncations, and point mutations [11]. LINE-1, as well as other transposable elements (TE), causes diseases through different mechanisms, such as insertional mutations, recombination, chromosomal rearrangements, expression, or epigenetic modifications [12].

An active LINE-1 sequence is approximately 6 Kb in size and encodes for two proteins, ORF1 and ORF2, the latter presenting with the enzymatic activity required for retrotransposition (endonuclease and retro transcriptase activity) [13]. A long 3′ poly-A tail is also present. Many LINE-1 sequences are 5′ truncated, due to a defective insertion process, and often present with an inversion involving a significant portion of the 5′ residual sequence. The LINE-1 insertion site is usually between short stretches of thymine and adenine residues (5′-TTTT/AA-3′) on the leading strand, which is recognized as the consensus cleavage site of the LINE-1 endonuclease (L1 END). During the insertion process, the poly-A tail pairs with the thymine residues of the cleavage site and the free 3′-OH of the cut sequence primes the polymerization of the new strand. The cleavage of the lagging DNA strand normally occurs a few base pairs downstream, causing target site duplication. Therefore, the inserted sequence is normally flanked by short direct repeats, 7–20 bp in size [14].

We report on a familial case of Neurofibromatosis type 1, including two siblings and their father. Standard laboratory analyses (NGS and MLPA on genomic DNA) were performed in the two sailings, failing to detect a pathogenic variant. A combined approach based on genomic analysis on gDNA (Optical Genome Mapping and Whole Genome Sequencing) and cDNA (specific long-range PCR and sequencing) was disclosed in both the insertion of a LINE-1 sequence within intron 15, altering the physiological splicing and resulting in skewed expression of the mutated allele. The father refused to be tested.

## 2. Materials and Methods

### 2.1. Clinical Description

We report on two siblings, affected by Neurofibromatosis type 1. Clinical diagnosis was achieved based on skin pigmentary lesions (cafe-au-lait spots, axillary and inguinal freckling) and positive family history, with their father having been previously diagnosed with NF1.

**Figure 1:**
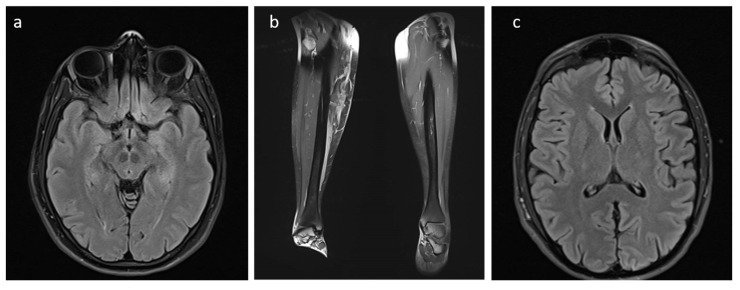
MRI of patients 1 (**a**,**b**) and 2 (**c**). Patient 1: Hyperintensity lesions with nonspecific significance on T2/FLAIR brain MRI in the thalamus (**a**). Presence of pathological tissue in the anterior compartment of the right leg. Patient 2: Hyperintensity areas on T2/FLAIR brain MRI in both lenticular capsule and thalamic regions (**c**).

Patient 1 is a 28-year-old woman, born from a bichorial twin pregnancy, her twin sister being unaffected. She presents widespread cafe-au-lait spots, axillary, inguinal, and neck freckling, subcutaneous neurofibromas of the trunk, right leg plexiform neurofibroma, and venous drainage abnormality in the corona radiata/centrum ovale. Hyperintensity lesions on T2/FLAIR brain MRI were observed in the thalamus, without phase-contrast impregnation and presenting with nonspecific significance (Figure 1). Legs MRI showed the presence of pathological tissue in the anterior compartment of the right leg. After the contrast medium, strengthening of the pathological tissue was noted, primarily in the medial and anterior portion of the tibia, for a stretch of approximately 5 cm. Small lesions were observed in the left leg, primarily involving the medial subcutaneous tissue. Some nodular images were observed in the context of the gemelli muscles. Because of the plexiform neurofibroma of the leg, the patient is currently being treated with selumetinib, an inhibitor of the mitogen-activated protein kinase 1 and 2 (MEK1/2) used for treating refractory fibromas in NF1 patients.

Patient 2 is a 23-year-old man who presented with widespread cafe-au-lait spots and axillary and inguinal freckling. Hyperintensity areas on T2/FLAIR brain and spinal MRI were observed in both lenticular capsule and thalamic regions, without significant postcontrast enhancement or diffusivity restriction (Figure 1). The course of the optic nerve exhibited abnormal curvature in the intraorbital tract, apparently without areas of altered signal or pathological phase-contrast impregnation.

### 2.2. gDNA and cDNA Extraction

Probands DNA was isolated from peripheral blood by a QIAsymphony automatic extractor (QIAGEN, Venlo, Netherlands). The TempusTM Spin RNA isolation kit (Applied Biosystem, Waltham, MA, USA) and cDNA Synthesis Kit (Euroclone, Pero, Italy) were used to isolate RNA from whole blood and for its reverse transcription, respectively. No sample was available for their affected father who refused to be tested.

### 2.3. Optical Genome Mapping (OGM) and Structural Variant Calling

A fresh blood aliquot from both siblings, collected in EDTA, was stored at −80 °C just after sampling. Ultra-high molecular weight (UHMW) DNA was extracted according to the manufacturer’s instructions (SP Frozen Human Blood DNA Isolation Protocol, Bionano Genomics, San Diego, CA, USA), and enzymatically labeled by the DLE-1 Enzyme (Bionano Prep Direct Label and Stain Protocol). Labeled DNA was loaded on the Saphyr chip and scanned on the Saphyr instrument (Bionano genomics, San Diego, CA, USA). The Saphyr chip was run to reach a minimum yield of 320 Gbp corresponding to 100X effective coverage. The de novo assembly and Variant Annotation Pipeline were executed on Bionano Solve software V3.6 using Human Genome Reference Consortium GRCh38 assembly as a reference for structural variants detection. Reporting and direct visualization of structural variants were performed on Bionano Access V1.6.

### 2.4. Whole-Genome Sequencing (WGS)

WGS was performed on genomic DNA in order to provide a fine characterization of the insertion event. Library preparation was carried out according to the manufacturer’s protocol from DNA PCR-Free Library Prep (Illumina) and sequenced on a NovaSeq6000 (Illumina) platform. The obtained NGS assay presented a mean coverage of 35X, with Q30 bases of around 87%. The TruSight Software Suite (illumina) and the integrated DRAGEN platform and IGV software were used for alignment, variant calling, and breakpoint data visualization. Sequencing data were aligned to the hg38 human reference genome.

### 2.5. Long-Range PCR and Sanger Sequencing

A long-range PCR was performed using the GoTaq^®^ Long PCR Master Mix (Promega, Madison, WI, USA) on both gDNA and cDNA. Primers were designed on exon 14 (forward sequence: AATCCAAGAAAACAG) and 17 (reverse sequence: AGTTCCCTTTTCCCT). PCR conditions on the gDNA were modified in order to obtain amplicons up to 10 Kb in size: 35 PCR cycles: 94 °C (30 s), 55 °C (30 s), and 65 °C (10 min). The extension time was reduced to 2 min when working on cDNA. The insertion was verified on gDNA by electrophoresis on a 1% agarose gel. The effect of the insertion was verified on cDNA by direct Sanger sequencing, using a Big Dye^®^ sequencing kit (Applied Biosystems, Foster City, CA, USA). Sequences were uploaded on an automated sequencer ABI Prism^®^ 3130 Xl Genetic Analyzer (Applied Biosystems) and the electropherograms were compared with the *NF1* wild-type sequence (NCBI GenBank accession n. NM_000267).

### 2.6. Quantitative Real-Time PCR

The commercially available TaqMan Gene Expression Assay (Applied Biosystems, Waltham, MA, USA) was performed for quantitative Real-Time PCR analysis of *NF1*, using a primer for the exon 1-2 junction. Human ACTB (Beta Actin) was used as an endogenous control.

## 3. Results

**Figure 2:**
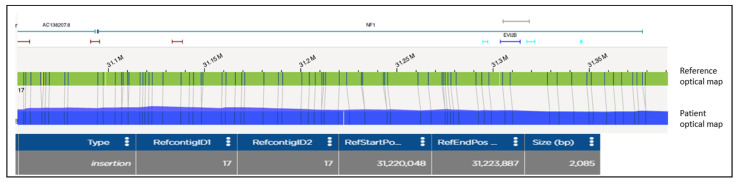
OGM analysis disclosing the presence of a 2.085 Mb insertion in *NF1* gene. The patient optical map (blue segment) is aligned and compared with the reference optical map (green segment). Molecular labels are reported as vertical lines on both patient and reference maps. In the patient’s map, the two labels at position [GRCh38] 17:31,220,048 and 17:31,223,887 present with a reciprocal distance of 5.924 Mb (instead of 3.839 Mb), showing a 2.085 Mb insertion also visible as an extra label (yellow). The gene content is shown at the top of the as a differently colored segments.

**Figure 3:**
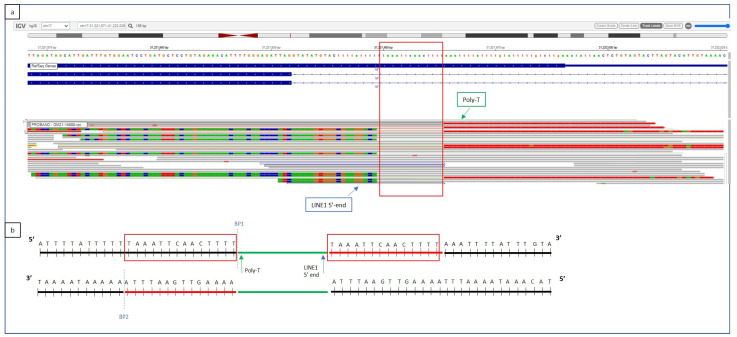
(**a**) Whole Genome Sequencing output from TruSight Software (Illumina). (**b**) Graphical representation of the LINE-1 insertion site. WGS shows the insertion of a poly-T segment within intron 15, 32 bases downstream of exon 15, corresponding to the 3′ poly-A end of the LINE-1 transposable element (green arrow). The 14-base duplicated region, normally located at both the extremities of the LINE-1 insertion, as a consequence of its integration mechanism, is reported as a red square. The first bases of the 5′ end of the inverted and truncated LINE-1 element are visible on WGS (**a**) as mismatched strings at the left side of the insertion (blue arrow). The inserted sequences are represented according to their base composition color (red for T, green for A, brown for G, blue for C).

Optical Genome Mapping analysis on gDNA from the affected siblings detected a 2.1 Kb insertion within the interval [GRChr38] 17q11.2(31,220,048-31,223,887) of the *NF1* sequence (Figure 2). The involved region was amplified by long-range PCR, and the insertion was visualized on an electrophoresis agarose gel, as an extra heavier band (wt allele 6Kb, alternative allele 8 Kb). In order to determine the origin of the inserted sequence and characterize the insertion site, WGS was utilized. The insertion of a highly repeated sequence, corresponding to a 5′truncated and inverted LINE-1 element (Long INterspersed Nuclear Elements), was identified within intron 15. The leading DNA strand cleavage occurred 34 bp downstream exon 15, where the consensus cleavage site 5′-TTTT/AA-3′ is present. LINE-1 direct integration starts with the polymerization, at the 5′ end of a poly-T stretch from the poly-A tail template (Figure 3). The 14 bp target sequence is repeated at both sides of the integrated region. The 3′ portion of the integrated sequence was also detected by WGS, which provided information about the last 372 nucleotides of the inserted element (Figure 3).

Long-range PCR on cDNA was performed using the same primers utilized for gDNA amplification and detected a weak and slightly shorter extra-band in siblings. Direct Sanger sequencing revealed a frameshift, consisting of the complete skipping of exon 15. The *NF1* transcript amount was evaluated by Real-Time PCR, which disclosed no difference between patients and unaffected controls (data not shown).

## 4. Discussion

We illustrate three cases of Neurofibromatosis type 1 in a single family, including the father and two siblings. Genomic analysis on gDNA and cDNA in siblings detected a 2.1 Kb insertion of part of the LINE-1 sequence within intron 15, which resulted in exon 15 skipping.

*NF1* gene is considered prone to transcriptional element insertions, likely due to its AT-reach sequence. Several cases of Alu insertion have been documented, but only a few reports of LINE-1 integration have been published, likely due to the difficulty of detecting large-sized (up to 6 kb) sequences using standard procedures [6,7]. In fact, specific PCR conditions must be set in order to amplify large amplicons. In addition, working on cDNA, reduced expression of the alternative allele is possible, due to the disruption of regulatory elements, non-sense mRNA decay, and skewed or monoallelic expression. cDNA analysis allows us to evaluate the transcript profile of a genomic aberration and, even if this information can be exhaustive from a diagnostic point of view, often does not provide insight into the molecular mechanism underlying the disease.

Three patients presenting with LINE-1 insertion in the *NF1* gene have been reported by Wimmer [7] and, in all of them, the rearrangement affected the physiological splicing. In two subjects, the insertion was detected within an exon, causing the skipping of the harboring exon (exon 23) in one of them and the exonization of a portion of the LINE-1 sequence (within exon 39) in the other. In the third patient, the LINE-1 insertion was detected within an intron (intron 9), causing the skipping of the preceding exon and the exonization of a short portion of the LINE-1 sequence.

In our patients, the insertion of the 5′truncated inverted LINE-1 element within intron 15 causes the skipping of the precedent exon, leading to an alternative transcript missing exon 15. LINE-1 insertion within introns results in a reduced expression, likely due to RNA polymerase II elongation defects or premature polyadenylation [15,16]. Accordingly, electrophoresis on cDNA reveals the presence of the alternative transcript as an extra weak and shorter band. These results suggest a skewing expression (with the normal allele increasing its transcription) rather than a non-sense mediated RNA decay (NMD). Skewed or monoallelic expression of the wild-type allele has been reported in patients harboring *NF1* pathogenic variants [6], likely reflecting the somatic nature of the disease, with localized clinical characteristics, usually associated with a second hit. The necessity of a biallelic inactivation, for the expression of some features such as café-au-lait spots, neurofibromas, tibial dysplasia, and malignant peripheral nerve sheath tumors [1], can also be considered when appraising the different clinical manifestations in our patients. In fact, the elder sister is more severely affected than her brother even if harboring the same constitutional event.

Exon skipping has been evaluated as a possible therapeutic approach in NF1, in order to minimize the pathogenic variant effects. In silico and in vitro tests have been performed in order to determine which exons could be skipped without affecting the neurofibromin expression and function [17]. The sole skipping of exon 15 produces a frameshift, leading to a truncated protein, as illustrated by our patients, literature reports [18,19], and cases noted in the LOVD (Leiden Open Variation Database, https://databases.lovd.nl, accessed on 1 July 2022) variant database, all of them presenting with the full-blown NF1 clinical phenotype. However, when both exons 15 and 16 are skipped, an in-frame deletion occurs, maintaining the protein function. A precise molecular diagnosis, as well as the evaluation of its consequences on the mRNA transcript, is crucial in paving the way for the development of targeted therapies.

## 5. Conclusions

Our results provide additional information on LINE-1 elements in NF1 molecularly undiagnosed patients and expand the mutational spectrum of the *NF1* gene, increasing the current knowledge about Neurofibromatosis type 1-associated mechanisms. Our data also highlight the importance of addressing patients who tested negative with standard procedures towards an alternative approach. Transcript sequencing still represents the gold standard for NF1 diagnosis, allowing the detection of intronic variants affecting the splicing, although allelic drop-out and skewed/monoallelic expression can lead to false negative results. At times, cDNA-based methods are unable to provide evidence for the causative genomic aberration, which represents crucial information for addressing proper genetic counseling, prenatal monitoring, and developing targeted therapy. Optical Genome Mapping detects structural rearrangements and large insertions on genomic DNA and may be regarded as an additional tool for increasing the diagnostic yield and expanding the current knowledge about the *NF1* gene.

## Data Availability

The data that support the findings of this study are available upon request from the corresponding author. The data are not publicly available due to privacy or ethical restrictions.
